# Endophilin-A1 BAR domain interaction with arachidonyl CoA

**DOI:** 10.3389/fmolb.2014.00020

**Published:** 2014-10-28

**Authors:** Maxim V. Petoukhov, Winfried Weissenhorn, Dmitri I. Svergun

**Affiliations:** ^1^Hamburg Unit, European Molecular Biology Laboratory c/o DESYHamburg, Germany; ^2^Unit of Virus Host Cell Interactions, University Grenoble AlpesGrenoble, France; ^3^Unit of Virus Host Cell Interactions, Centre National de la Recherche ScientifiqueGrenoble, France

**Keywords:** endophilin, BAR domain, saturated fatty acid, membrane curvature, endocytosis, solution scattering, molecular modeling

## Abstract

Endophilin-A1 belongs to the family of BAR domain containing proteins that catalyze membrane remodeling processes via sensing, inducing and stabilizing membrane curvature. We show that the BAR domain of endophilin-A1 binds arachidonic acid and molds its coenzyme A (CoA) activated form, arachidonyl-CoA into a defined structure. We studied low resolution structures of endophilin-A1-BAR and its complex with arachidonyl-CoA in solution using synchrotron small-angle X-ray scattering (SAXS). The free endophilin-A1-BAR domain is shown to be dimeric at lower concentrations but builds tetramers and higher order complexes with increasing concentrations. Extensive titration SAXS studies revealed that the BAR domain produces a homogenous complex with the lipid micelles. The structural model of the complexes revealed two arachidonyl-CoA micelles bound to the distal arms of an endophilin-A1-BAR dimer. Intriguingly, the radius of the bound micelles significantly decreases compared to that of the free micelles, and this structural result may provide hints on the potential biological relevance of the endophilin-A1-BAR interaction with arachidonyl CoA.

## Introduction

Endophilin-A1 belongs to the superfamily of Bin-Amphiphysin-Rvs (BAR) domain-containing proteins that have been implicated in the generation of membrane curvature by deforming membranes into tubular structures (Farsad et al., 2001; Dawson et al., 2006). BAR domains play an important role during endocytosis, underlined by the essential role of endophilin-A1 for the formation of synaptic vesicles at the plasma membrane (Itoh and De Camilli, 2006).

The canonical BAR domain structure adopts a crescent shaped dimeric conformation with (N-Bar) or without (Bar) an N-terminal amphipathic helix that folds upon membrane contact (Peter et al., 2004; Weissenhorn, 2005; Gallop et al., 2006; Masuda et al., 2006; Wang et al., 2008). A subset is connected to membrane interacting PH (Pleckstrin Homology) domains or PX (Phox Homology) domains with binding specificities for phosphoinositides (Li et al., 2007; Pylypenko et al., 2007; Zhu et al., 2007; Wang et al., 2008). The superfamily extends to F-BAR (FCH-BAR) (Itoh et al., 2005; Shimada et al., 2007) and I-BAR (Inverse BAR) domains (Millard et al., 2005; Saarikangas et al., 2009), which use similar structures to bend membranes or to stabilize flat membranes.

Several principles govern the generation of membrane curvature employed to form narrow tubes and small vesicles. Firstly, N-BAR domains use partial insertion of helical segments into the bilayer to displace lipids thus acting like a wedge (Itoh and De Camilli, 2006; Cui et al., 2013), which can induce membrane fission (Boucrot et al., 2012). Secondly, the curved structure itself induces/stabilizes membrane curvature (Itoh and De Camilli, 2006). Thirdly, helical polymerization might be a common mechanism for all BAR members to provide a membrane-bound protein scaffold with an intrinsic BAR domain-type curvature (Frost et al., 2008; Mim et al., 2012). A fourth component, the specific role of lipids during endocytosis is less clear with exception of a functional role for phosphatidylinositols (Rohrbough and Broadie, 2005). Although endophilin-A1 has been originally implicated in lipid modification (Schmidt et al., 1999), such a role was not confirmed (Gallop et al., 2005).

Small-angle X-ray scattering (SAXS) is an increasingly popular method allowing to study the structure, folding state and flexibility of native particles and complexes in solution and to rapidly analyze structural changes in response to variations in external conditions (Svergun et al., 2013). In the present work synchrotron SAXS has been employed for structural analysis of free endophilin-A1-BAR and its interactions with arachidonyl-CoA. The oligomerization mechanisms of endophilin-A1-BAR are elucidated and structural models of individual arachidonyl-CoA micelles are constructed. The dimeric endophilin-A1-BAR is found to bind two arachidonyl-CoA micelles and the structure of the complex is reconstructed from the SAXS data. Although the original proposition that endophilin-A1 uses arachidonyl-CoA as a substrate (Schmidt et al., 1999) has not been validated, it is intriguing that endophilin-A1-BAR forms a defined complex with arachidonic acid and its coenzyme A (CoA) activated form, arachidonyl-CoA.

## Materials and methods

### Protein expression and purification

Endophilin-A1-BAR (residues 1–256 of mouse endophilin-A1) was expressed and purified as described (Weissenhorn, 2005). Briefly, endophilin-A1-BAR was expressed in *E. coli* cells BL21 codon plus™ (Invitrogen). The bacterial cell lysate was applied onto Ni-chelating sepharose (GE Healthcare),washed extensively with buffer A (50 mM Tris-HCl pH 8, 0.3 M NaCl) supplemented with 1 M NaCl, followed by a buffer A wash supplemented with 1 M KCl to remove nucleic acids. In order to remove lipids potentially associated with endophilin the column was washed with buffer A supplemented with 1% CHAPS. Finally any non-specific contaminants were removed with a buffer A wash supplemented with 0.1 M imidazole. The protein was eluted with 0.3 M imidazole in buffer A. A final gel filtration was performed on a Superdex 200 column (GE Healthcare) in buffer B (20 mM Bicine pH 9, 100 mM NaCl).

### Native page and size exclusion chromatography (SEC)

25 μl of endophilin-A1-BAR (10 μM) in buffer B were mixed with arachidonyl-CoA (2 mM) solubilized in H_2_O or arachidonic acid solubilized in ethanol, incubated on ice for 30 min prior to separation by native PAGE. 3 μl of ethanolic stock solutions of arachidonic acid concentrations were incubated with 150 μl of buffer B and the turbidity was recorded at 600 nm. The concentration at which the absorption changed in a sigmoidal manner was determined as the critical micelle forming concentration (CMC). For SEC analysis of endophilin, endophilin was separated on a superdex 200 column at a concentration of 100 μM in buffer B and 100 μM of endophilin were mixed with arachidonyl-CoA (2 mM) and incubated on ice prior to SEC in buffer B. The elution profile was monitored at 280 nm.

### SAXS data collection and processing

All experiments were performed at beamline X33 of EMBL (DESY, Hamburg) using a MAR345 image plate detector (experimental parameters are summarized in Table 1). At a sample-detector distance of 2.7 m, the range of momentum transfer 0.1 < s < 5 nm^−1^ was covered (*s* = 4π sin(θ)/λ, where 2θ is the scattering angle and λ = 0.15 nm is the X-ray wavelength). First, arachidonyl-CoA was measured at different concentrations (0.1, 0.25, 0.5, 1, 2, 4, 8 mg/ml) in buffer B to determine the size of arachidonyl-CoA micelles. Endophilin-A1-BAR was measured at several solute concentrations ranging from 2 to 10 mg/ml to assess the concentration dependence. Finally, arachidonyl-CoA was titrated at concentrations of 0.1, 0.2, 0.5, 1, 2, 2.5, 3, 4, 5, 6, 8 mg/ml into endophilin-A1-BAR solutions at 1, 2, 3.25, and 4 mg/ml. The scattering profiles recorded for all samples were normalized to the solute concentration and the data processing was performed using PRIMUS (Konarev et al., 2003). The forward scattering *I*(*0*) and the radii of gyration *R*_g_ were evaluated using the Guinier approximation (Guinier, 1939), assuming that at very small angles (*s* < 1.3/*R*_g_), the intensity is represented as *I*(*s*) = *I*(0) *exp*( − (*sR*_g_)^2^/3). The maximum dimensions *D*_max_ were computed using the indirect transform package GNOM (Svergun, 1992), which also provides the distance distribution function *p(r)*. The molecular masses (MM) of the solutes were estimated from SAXS data by comparison of the forward scattering with that from reference solutions of bovine serum albumin. The contrast of the micelles is higher than that of the protein (the average electron density of the CoA micelles computed from their chemical composition is 560 e/nm^3^ and that of the protein is 440 e/nm^3^), and the computed MM of the micelles was appropriately corrected. The hydrated volumes of the particles *V*_p_ were estimated using the Porod invariant (Porod, 1982) as an additional check of oligomeric state/stoichiometry (*V*_p_ in nm^3^ is typically 1.5–2 times larger than MM in kDa).

**Table 1:** **Data-collection and scattering-derived parameters**.

	**Endophilin-A1**	**Arachidonyl-CoA**	**Complex**
**DATA-COLLECTION PARAMETERS**			
Beam line	X33 (DORIS III)	X33 (DORIS III)	X33 (DORIS III)
Beam geometry	2 × 0.6 mm^2^	2 × 0.6 mm^2^	2 × 0.6 mm^2^
Wavelength (nm)	0.15	0.15	0.15
*s* range (nm^−1^)	0.1–5.0	0.1–5.0	0.1–5.0
Exposure time (s)	120	120	120
Concentration range (mg/mL)	2–10	0.1–8	2–8
Temperature (K)	283	283	283
**STRUCTURAL PARAMETERS**			
MM, kDa	50 ± 10	60 ± 10	130 ± 20
*R*_*g*_, nm	3.3 ± 0.1	3.4 ± 0.1	5.9 ± 0.2
*D*_*max*_, nm	13.5 ± 0.5	8.5 ± 0.5	19.0 ± 1
*V*_*p*_, nm^3^	90	210	480
χ	1.59	1.53	2.59
**SOFTWARE EMPLOYED**			
Primary data processing	PRIMUS	PRIMUS	PRIMUS
*Ab initio* analysis	DAMMIN	DAMMIN	DAMMIN/MONSA
Computation of model intensities	CRYSOL	CRYSOL	CRYSOL
Analysis of oligomeric equilibrium	OLIGOMER	–	–
Hybrid modeling	CORAL/BUNCH	SASREF	SASREF
Superposition and averaging	SUPCOMB/DAMAVER	SUPCOMB/DAMAVER	SUPCOMB/DAMAVER

The low resolution shapes of the species were determined by the *ab initio* modeling program DAMMIN (Svergun, 1999), which represents a molecule as assembly of beads inside a spherical search volume of the diameter *D*_max_. Starting from a random model, DAMMIN employs simulated annealing (SA) to build a compact interconnected assembly fitting the experimental data *I*_exp_(*s*) to minimize the discrepancy:

$$\chi^{2}=\frac{1}{N-1}\sum_{j}{\left[\frac{I_{\exp}\left(s_{j}\right)-cI_{calc}\left(s_{j}\right)}{\sigma \left(s_{j}\right)}\right]}^{2}$$

where *N* is the number of experimental points, *c* a scaling factor and *I*_*calc*_(*s*_*j*_) and σ(*s*_*j*_) are the calculated intensity and the experimental error at the momentum transfer *s*_*j*_, respectively. The multiphase extension of DAMMIN, developed for the modeling of multi-component particles by simultaneously fitting several scattering profiles, MONSA (Svergun and Nierhaus, 2000; Petoukhov and Svergun, 2006) was employed in the present study for structural investigation of the endophilin-A1-BAR–arachidonyl-CoA complexes. The protein and micelle parts were represented by two phases and the two scattering curves (unbound endophilin-A1-BAR and the protein-micelle complex) were fitted simultaneously.

The oligomeric equilibrium of endophilin-A1 was analyzed by OLIGOMER (Konarev et al., 2006), which fits the experimental data from a mixture by a linear combination of the scattering profiles from its components, weighted by their volume fraction in the mixture.

The scattering intensities from the atomic models were computed by the program CRYSOL (Svergun et al., 1995), which either predicts theoretical scattering patterns or fits the experimental data by adjusting the excluded volume and the contrast of the hydration layer. Programs BUNCH (Petoukhov and Svergun, 2005) and CORAL (Petoukhov et al., 2012) were applied to add missing terminal portions to the available high resolution model of the dimeric endophilin-A1-BAR (Weissenhorn, 2005, Protein Data Bank entry 1ZWW). The two programs employ SA based search of the optimal spatial arrangement and possible conformations of missing loops represented as interconnected chains of dummy residues (Petoukhov et al., 2002) attached to the appropriate residues in the domains of known structure.

Rigid body modeling of arachidonyl-CoA micelles and of the protein-micelle complex was performed by SASREF (Petoukhov and Svergun, 2005). In the former case, atomic coordinates of an arachidonyl-CoA monomer were used, in the latter case, one endophilin-A1-BAR dimer and two intact arachidonyl-CoA micelles were treated as rigid bodies. Their mutual positions and orientations were optimized by SA to build interconnected arrangement without steric clashes yielding the best fit to the SAXS data.

Superposition and averaging of the models were done using the programs SUPCOMB (Kozin and Svergun, 2001) and DAMAVER (Volkov and Svergun, 2003), respectively.

## Results

### Endophilin-A1 BAR interacts with arachidonyl-CoA

**Figure 1:**
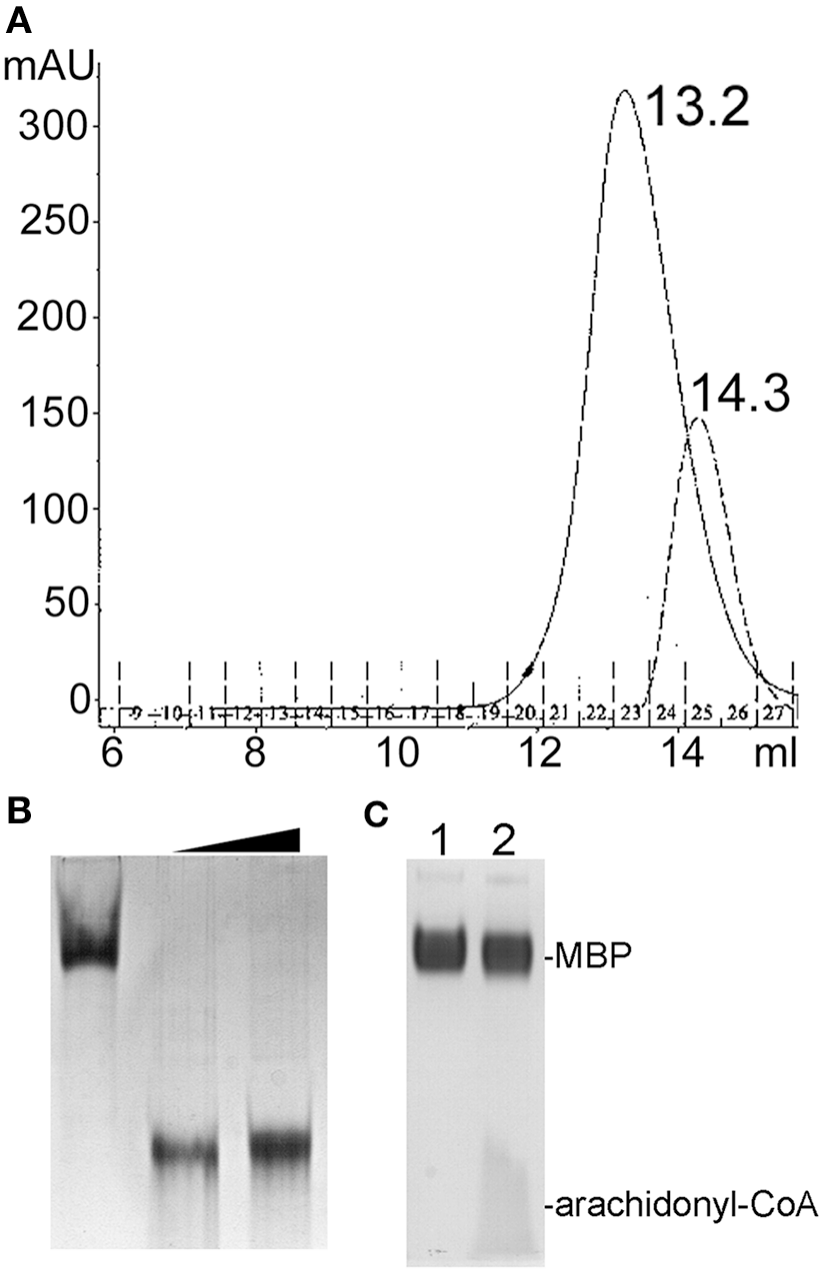
**(A)** Size exclusion chromatography profiles of endophilin-A1-BAR with 2 mM arachidonyl-CoA (peak at 13.2 ml) and without (peak at 14.3 ml) arachidonyl-CoA. **(B)** Native gel electrophoresis of endophilin-A1-BAR (lane 1) incubated with arachidonyl-CoA concentrations of 2 mM (lanes 2) and 10 mM (lanes 3). **(C)** No gel shift was observed with the control protein MBP (lane 1) incubated with arachidonyl-CoA (lane2).

**Figure 2:**
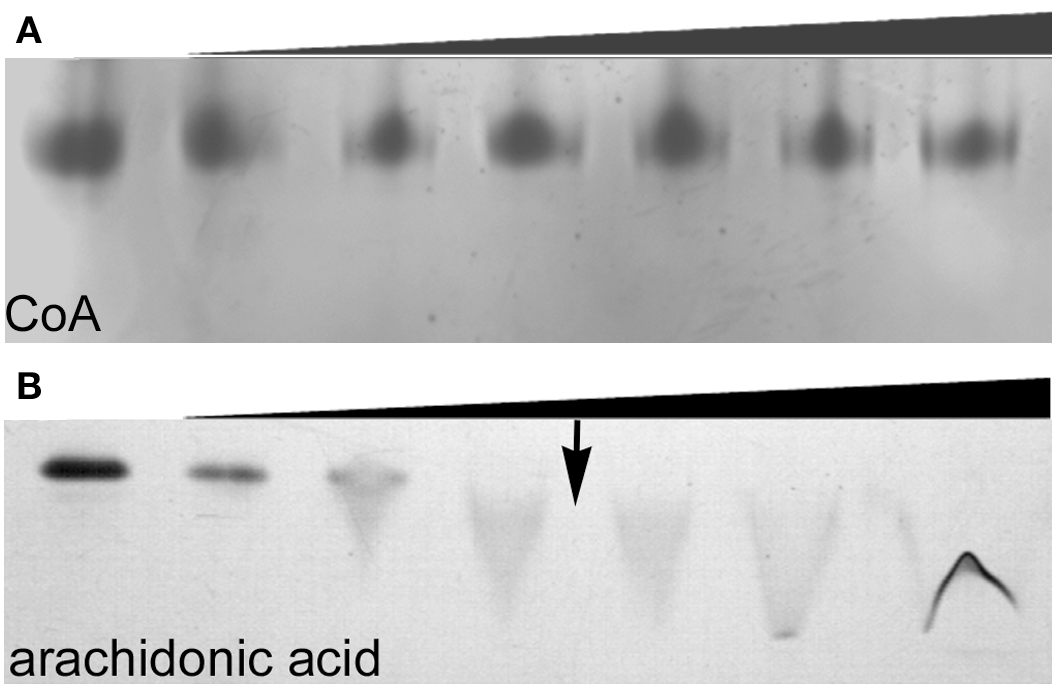
**Native gel electrophoresis of endophilin-A1-BAR at a concentration of 10 μM incubated with (A) coenzyme A at 0, 0.02, 0.2, 0.5, 1, 2, 4 mM concentrations and (B) arachidonic acid (0, 0.2, 0.5, 1, ↓ 1.5, 2, 2.5 mM)**. The transition to a critical micelle concentration is indicated by an arrow (↓).

Size exclusion chromatography (SEC) of endophilin-A1-BAR revealed an elution peak at 14.3 ml from a superdex 200 column. When incubated with arachidonyl-CoA the endophilin-A1-BAR peak broadens and shifts to 13.2 ml indicating complex formation (Figure 1A). Interaction between endophilin-A1-BAR and arachidonyl-CoA was further confirmed by native gel electrophoresis, showing a single band for endophilin-A1-BAR and a unique band appearing upon complex formation with arachidonyl-CoA, which migrates further into the gel (Figure 1B). The control experiment with CoA alone demonstrated that endophilin-A1-BAR does not interact with increasing concentrations of CoA (Figure 2A). The lack of complex formation between endophilin-A1-BAR and CoA was further confirmed by isothermal titration calorimetry (data not shown). In contrast, clear band shifts were observed upon incubation of endophilin-A1-BAR with arachidonic acid (C_20:4_) (Figure 2B), indicating that the unsaturated lipid is the main determinant of interaction.

### Small angle X-ray scattering (SAXS)

**Figure 3:**
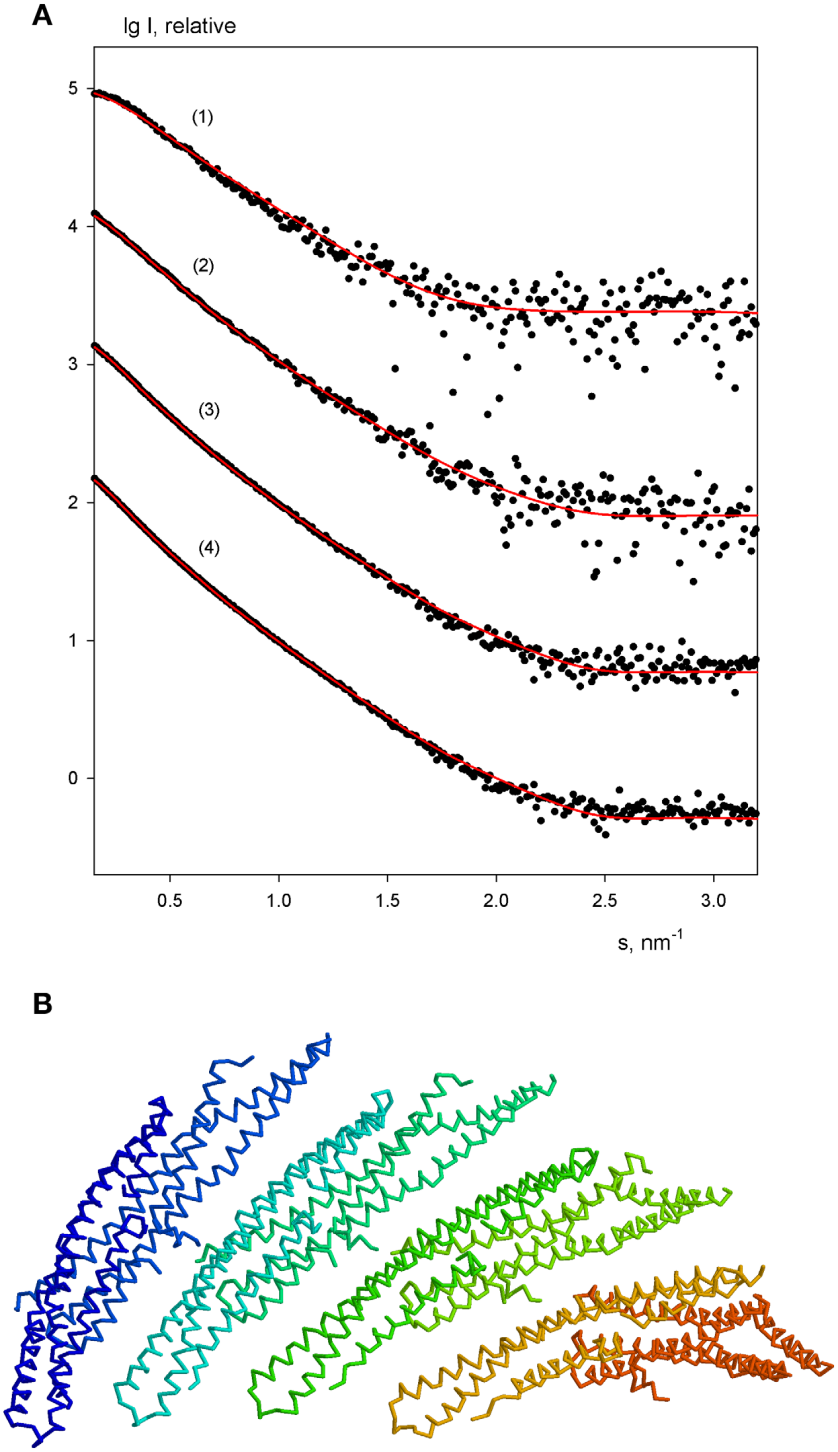
**Oligomeric equilibrium of endophilin-A1. (A)** SAXS profiles at increasing protein concentrations (*c* = 2, 3, 4, and 6 mg/ml). Experimental data are denoted by black dots, fits by oligomeric mixtures are shown as red solid lines. **(B)** Structural model of the putative octamer with helical symmetry built from dimeric endophilin-A1-BAR.

The experimental scattering patterns from free endophilin-A1-BAR at different concentrations are presented in Figure 3A. The data demonstrated strong concentration dependence (the obtained *R*_*g*_ and *I*_0_ values are summarized in Table 2). The molecular mass (MM) estimated from the forward scattering of the lowest concentration (*c* = 2 mg/ml) and the value of the hydrated particle volume (Table 1) point to a dimeric state of the solute (the calculated MM of the monomer is 29 kDa). The increases of *R*_*g*_ and *I*_0_ values with increasing protein concentrations point to formation of higher order oligomers of endophilin-A1-BAR. Such oligomeric behavior is not unexpected for endophilin-A1-BAR given that electron microscopy (EM) studies reported its propensity to form helical assemblies upon interactions with membranes (Frost et al., 2008; Mim et al., 2012). In order to quantify the observed multimerization phenomenon at higher protein concentrations, a putative octamer (Figure 3B) with helical symmetry has been built based on the dimeric model. The program OLIGOMER (Konarev et al., 2006) was then applied to find the volume fractions of the octamer and its subcomplexes (dimers, tetramers and hexamers) minimizing the discrepancy between the experimental data and the scattering intensity computed for the mixture. Endophilin hexamers were not required to fit the data and the good fits could be obtained by three-component mixtures (Figure 3A). The obtained volume fractions of the dimer, tetramer and octamer (Table 2) indicate that the dimer stays a predominant species at intermediate concentrations while the proportion of octamers increases with concentration so that the octamers may constitute the precursors for the formation of the large helical structures observed by EM.

**Table 2:** **Oligomeric equilibrium of free endophilin sample**.

**Conc., mg/ml**	**R_g_, nm**	**I0, arb. units**	***V*^dimer^_fr_**	***V*^tetramer^_fr_**	***V*^octamer^_fr_**
2	3.3	100	1.0	–	–
3	4.2	130	0.61	0.17	0.22
4	4.6	160	0.51	0.14	0.35
6	4.7	170	0.52	0.03	0.45

**Figure 4:**
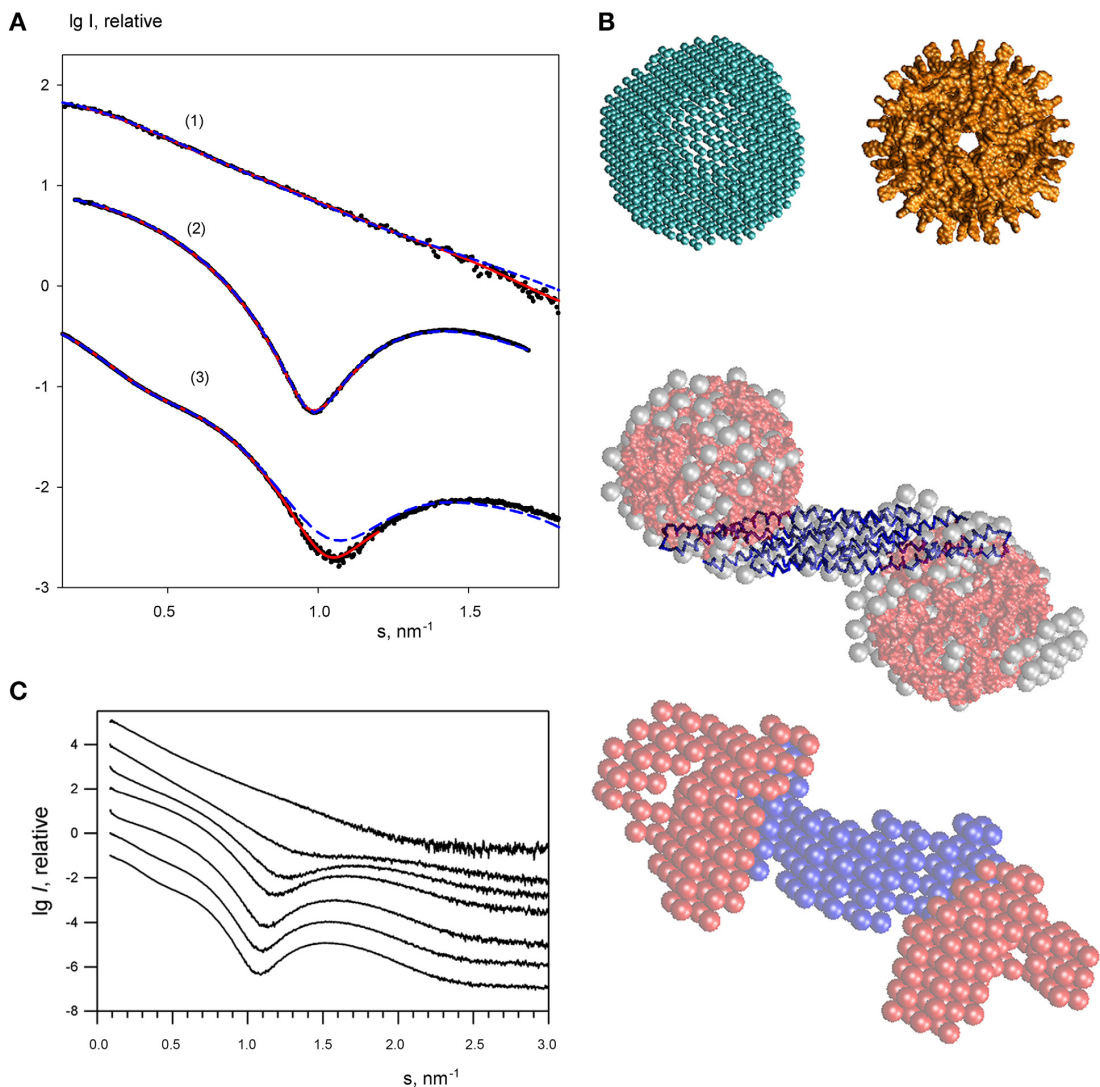
**Small angle X-ray scattering results for the endophilin-A1-BAR complex with arachidonyl-CoA micelles. (A)** Scattering profiles of free endophilin-A1-BAR at *c* = 2 mg/ml (1), arachidonyl-CoA micelles at *c* = 1 mg/ml (2) and their complex at *c* = 3.5 mg/ml (3). Experimental data are denoted by black dots, fits from the *ab initio* and atomic structure-based models are shown as red solid and blue dashed lines, respectively. The curves are displaced in logarithmic scale for better visualization. **(B)** Structural models. Top panel: *ab initio* DAMMIN model of the arachidonyl-CoA micelle (left) and the one built from the atomic structure of monomers using icosahedral symmetry (right). Middle panel: *ab initio* DAMMIN model of the complex (gray beads) overlaid with rigid body model (endophilin is shown as blue backbone, two arachidonyl-CoA micelles are shown in red). Bottom panel: Multiphase bead model of the complex reconstructed by MONSA by simultaneous fitting of two scattering patterns. **(C)** The scattering profile of endophilin-A1-BAR at 4 mg/ml (top) changes upon titration of increasing concentrations of arachidonyl-CoA (0, 1, 2, 3, 4, 6, 8 mg/ml, from top to bottom).

For the lowest endophilin concentration (where the solute was proven to be monodisperse) multiple *ab initio* reconstructions were performed by DAMMIN (Svergun, 1999) with no symmetry restrictions and assuming two-fold symmetry. Individual reconstructions produced similar elongated shapes neatly fitting the experimental profile (Figure 4A). The *ab initio* bead models are also in good agreement with the crystallographic model (PDB entry 1ZWW) of endophilin-A1-BAR. The scattering curve computed from the high resolution model provides a reasonable fit to the experimental data (Figure 3A) showing only minor deviations, which may be attributed to the fact that the N-terminal 30 residues are missing in the atomic structure. Addition of the missing N-terminal part to the crystallographic dimer allowed to further improve the fit yielding a discrepancy χ = 1.53 (Figure 4A). This confirms that endophilin-A1-BAR is dimeric in solution at low concentrations and adopts the structure similar to the crystallographic one (Weissenhorn, 2005).

The scattering curves from arachidonyl-CoA were then recorded at different concentrations revealing arachidonyl-CoA micelles at concentrations above 0.1 mg/ml. A typical scattering curve (Figure 4A) demonstrates profound first minimum and maximum characteristics for globular particles. Indeed, the shape reconstructions by DAMMIN were similar to hollow spheres with the outer and inner radii of 4.0 and 2.5 nm, respectively (Figure 4B, top panel). The inner cavity corresponds to the hydrophobic tails of arachidonyl-CoA whose contrast to the solvent is much lower than that of the hydrophilic heads. A putative model of an arachidonyl-CoA micelle was built using SASREF (Petoukhov and Svergun, 2005) from monomers of arachidonyl-CoA replicated assuming icosahedral symmetry whereby the heads formed the surface and the tails comprised the core of the micelle. This model having the radius of 4.0 nm agreed well with the typical *ab initio* shape (Figure 4B, top panel) and provided a good fit to the experimental SAXS curve with χ = 1.59 (Figure 4A). The MM of such a micelle was estimated to be approximately 60 kDa.

In order to study the interaction of endophilin-A1-BAR with arachidonyl-CoA the protein solution was titrated with arachidonyl-CoA at ratios varying from 8:1 to 1:5, resulting in significant changes in the experimental SAXS profiles (representative curves are given in Figure 4C, and the entire set of data in Supplementary Figure 1). During titration, the stoichiometric composition should be the one yielding the maximum apparent size of the solute (maximum amount of complex and minimum amount of individual components). The largest solute size was detected from the scattering profile at a concentration ratio (protein:arachidonyl-CoA) of 1:2.5 yielding *R*_g_ = 5.9 nm and *D*_max_ = 19 nm. Given that the MM of the endophilin monomer is 29 kDa and that of the CoA micelle is about 60 kDa, this ratio corresponds to a slight molar excess of CoA (1.3 CoA micelles per endoplilin monomer). The result suggesting the binding of two arachidonyl-CoA micelles to the endophilin-A1-BAR dimer is also in agreement with the change of the estimated MM and the Porod volume (Table 1). The scattering from the complex (Figure 4A, curve 3) has a very characteristic appearance, and displays a maximum at s_1_ ≈ 0.75 nm^−1^ indicative of the presence of two distinct domains with an interdomain distance of approximately 2π/s_1_ ≈ 8.5 nm. As described in Methods, the contrast of the micelles is higher than that of the protein and the scattering from the complex is largely defined by that of the arachidonyl-CoA moiety. The interdomain distance should therefore correspond to the separation between the arachidonyl-CoA micelles. Moreover, the minimum of curve 3 at s_2_ ≈ 1.1 nm^−1^, which is due to the spherical shape of the micelles, is shifted toward larger angles compared to the scattering by the free micelles (where the minimum was at ≈ 1.0 nm^−1^). This result indicates that the micelles are shrunk by about 10% in size upon complex formation. These qualitative considerations were further corroborated by the *ab initio* shape modeling of the complex (a typical fit is presented in Figure 4A). The low resolution models built by DAMMIN demonstrate a dumbbell shape whereby two quasi-spherical peripheral portions are connected by a rod-like middle portion (Figure 4B, middle panel). Moreover, multiphase bead modeling with MONSA (Petoukhov and Svergun, 2006) allowed distinguishing the protein component from the micelles by simultaneous fitting the scattering curves of unbound protein and the protein/micelle complex. A typical model generated by MONSA (Figure 4B, bottom panel) shows that each arm of the endophilin-A1-BAR dimer interacts with one arachidonyl-CoA micelle. In the MONSA model, the averaged dimensions of the micelles bound to endophilin-A1-BAR are about 10% smaller than those in the unbound state.

To provide a more detailed structural characterization of the complex, rigid body modeling has been applied. First, a tentative model of ~10% shrunk arachidonyl-CoA micelle with a radius of 3.4 nm has been generated using icosahedral symmetry, then SASREF has been employed to symmetrically position two copies of such a micelle with respect to the endophilin-A1-BAR dimer by fitting the experimental SAXS data. The resulting fit with χ = 2.59 (Figure 4A) appears rather reasonable given that the tentative structures of the two micelles were used as rigid bodies and were not further refined. The location of the two micelles obtained by SASREF at the peripheral arms of the protein (Figure 4B, middle panel) further corroborated the conclusions from *ab initio* reconstructions about stoichiometry and overall architecture of the complex.

## Discussion

Endophilin-A1-BAR is essential for endocytosis including the formation of synaptic vesicles from the plasma membrane, where it plays a major role in inducing and stabilizing membrane curvature (Itoh and De Camilli, 2006). In the present study we demonstrate that at low concentrations endophilin-A1-BAR stays dimeric in solution with the structure similar to that of the crystallographic dimer (Weissenhorn, 2005). At the solute concentrations above 4 mg/ml endophilin-A1-BAR shows a propensity to form larger oligomers. Our data point to formation of side-by-side helically arranged octamers, which are in equilibrium with the dimers and tetramers. These higher oligomers resemble the repeating units reported earlier in the EM studies (Frost et al., 2008; Mim et al., 2012) and our results indicate that the propensity of endophilin-A1-BAR to form helical multimers exists not only for the membrane-bound form but also for the free protein in solution.

We demonstrate that endophilin-A1-BAR interacts with the unsaturated lipid arachidonic acid and its coenzyme A activated form *in vitro.* The structural model obtained from SAXS reveals two arachidonyl-CoA micelles assembled at the distal ends of the dimeric BAR domain. The radius of unbound arachidonyl-CoA micelles is ~10% larger than that of the micelles bound to endophilin-A1-BAR indicating that the endophilin-A1-BAR interaction with the lipid micelle rendered the micelle more compact. Although the micelles are located at the distal end of the two endophilin arms, we cannot exclude the possibility that the presence of the amphipathic helix might have contributed to the micelle shrinkage. We show further by native gel analysis that the interaction depends on the unsaturated acyl chain and not on the CoA moiety as reported previously (Gallop et al., 2005). Although the significance of endophilin-A1-BAR interaction with arachidonyl-CoA (Schmidt et al., 1999) was questioned (Gallop et al., 2005), our data provide a clear evidence for a specific interaction of endophilin-A1-BAR with the lipid and its CoA activated form. The lipid micelle interaction site on endophilin-A1-BAR overlaps with the proposed position of the SH3 domain that was also mapped to the distal tips of a BAR domain dimer (Wang et al., 2008).

Although the physiological relevance of the endophilin-A1-BAR complex with arachidonyl-CoA is not clear, the complex is surprisingly stable producing reproducibly a single band on a native gel and high quality X-ray scattering data. These observations together with the shrinkage of the micelles indicate a well-defined complex and specific interactions between endophilin-A1-BAR and the lipid. Unsaturated fatty acids are highly enriched in synaptic membranes (Takamori et al., 2006). They have been reported to promote endocytosis (Ben Gedalya et al., 2009) and affect the localization of synaptojanin (Marza et al., 2008), an interaction partner of endophilin-A1-BAR (Gad et al., 2000). Furthermore, lipid unsaturation affects endocytotic sorting of lipids (Marza et al., 2008) and lipidic cleavage products influence endocytosis (Brown et al., 2003; Staneva et al., 2004; Rohrbough and Broadie, 2005). Given the intriguingly regular structure of the endophilin-A1-BAR arachidonyl-CoA complex further experiments are required to prove a link of endophilin to lipid metabolism *in vivo*.

Finally, we would like to stress that the SAXS analysis of protein systems in the presence of lipids is usually notoriously difficult because of problems in accounting for the signal from micelles. In the present study, thanks to the specificity of the complex and to the highly pure sample preparations, it was possible to model and explicitly represent the micellar contribution and to further utilize hybrid modeling of the protein-lipid complex. The methodology developed may become useful for other SAXS studies of protein-lipid interactions.

### Conflict of interest statement

The authors declare that the research was conducted in the absence of any commercial or financial relationships that could be construed as a potential conflict of interest.

## Abbreviations

BAR: Bin-Amphiphysin-Rvs domain-containing protein
SAXS: small-angle X-ray scattering
SEC: size exclusion chromatography
CoA: coenzyme A.
