# Supplementary figures and images for: Endophilin-A1 BAR domain interaction with arachidonyl CoA
Source: Front Mol Biosci. 2014 Oct 28;1:20. doi: 10.3389/fmolb.2014.00020 (PMC4428356; doi:10.3389/fmolb.2014.00020)

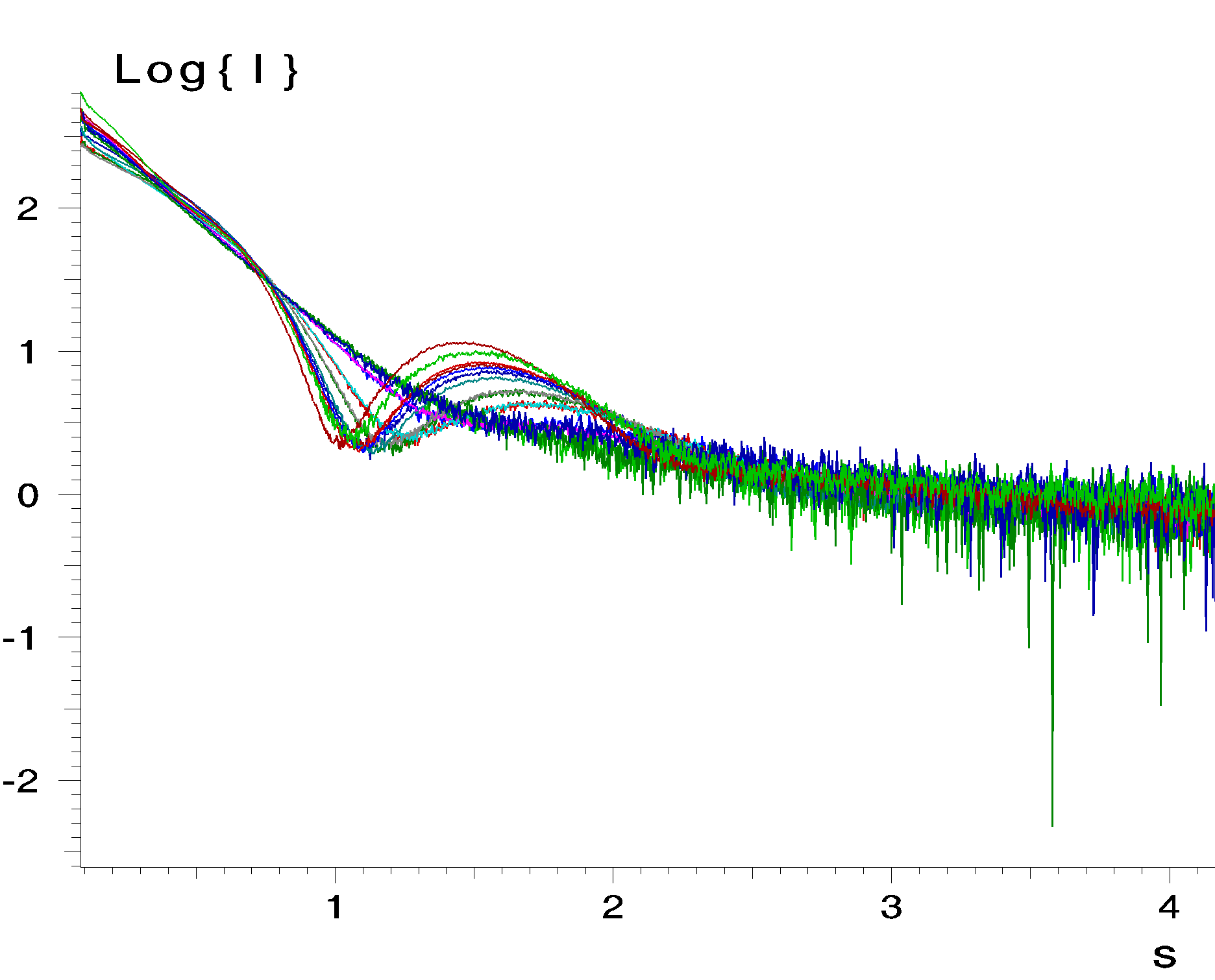

Supplement: Supplementary file 1 [file Image1.TIF]
